# Patient navigation to improve diabetes outpatient care at a safety-net hospital: a retrospective cohort study

**DOI:** 10.1186/s12913-017-2700-7

**Published:** 2017-11-21

**Authors:** Michal Horný, Wiljeana Glover, Gouri Gupte, Aruna Saraswat, Varsha Vimalananda, James Rosenzweig

**Affiliations:** 1 Department of Radiology and Imaging Sciences, Emory University School of Medicine, Woodruff Memorial Research Building, Room 1215A, 101 Woodruff Circle, Atlanta, GA 30322 USA; 2 Department of Health Policy and Management, Emory University Rollins School of Public Health, 1518 Clifton Road NE, Atlanta, GA 30322 USA; 3 Department of Health Law, Policy and Management, Boston University School of Public Health, 715 Albany St, Boston, MA 02118 USA; 4 Department of Technology, Operations, and Information Management, Babson College, 231 Forest Street, Babson Park, MA 02457 USA; 5 Cambridge Health Alliance, 1035 Cambridge Street, Cambridge, MA 02139 USA; 6 Tufts Medical Center, 800 Washington Street, Boston, MA 02111 USA; 7 Department of Medicine, Boston University School of Medicine, 715 Albany St, Boston, MA 02118 USA; 8 Center for Healthcare Organization and Implementation Research, Edith Nourse Rogers Memorial Veterans Affairs Medical Center, 200 Springs Road, Bedford, MA 01730 USA; 9 Hebrew Rehabilitation Hospital, 1200 Centre Street, Boston, MA 02131 USA

**Keywords:** Patient navigation, Diabetes management, Patient-centered medical home

## Abstract

**Background:**

Recent emphasis on value based care and population management, such as Accountable Care Organizations in the United States, promote patient navigation to improve the quality of care and reduce costs. Evidence supporting the efficacy of patient navigation for chronic disease care is limited. The objective of this study was to evaluate the effect of a patient navigation program on medical and administrative outcomes among patients with diabetes in an urban, safety-net hospital clinic setting.

**Methods:**

A retrospective cohort study with pre- and post-intervention periods was conducted. Eligible patients were those with A1C ≥ 8.5% and at least one appointment no-show in the previous 12 months. The intervention and reference groups were balanced on observed characteristics and baseline outcome levels using propensity score matching. The effect of patient navigation was isolated using the difference-in-differences approach. Primary outcomes were A1C, low-density lipoprotein cholesterol, triglycerides, random urine microalbumin, the number of scheduled appointments, clinic visits, emergency visits, and inpatient stays, and the percentage of arrivals, cancellations, and no-shows to scheduled appointments.

**Results:**

Of 797 eligible patients, 328 entered the navigation program. Matching reduced the sample size to 392 individuals (196 in each group). Patient navigation resulted in improved A1C (−1.1 percentage points; *p* < .001), more scheduled appointments (+ 5.3 per year; *p* < .001), more clinic visits (+6.4 per year; *p* < .001), more arrivals to scheduled appointments (+7.4 percentage points; *p* = .009) and fewer no-shows (−9.8 percentage points; *p* < .001).

**Conclusions:**

Navigation was associated with improved glycemic control and better clinic engagement among patients with diabetes. Further research is important to identify what features of navigation in diabetes care are critical to achieving success and to understand navigators’ role in other settings.

**Electronic supplementary material:**

The online version of this article (10.1186/s12913-017-2700-7) contains supplementary material, which is available to authorized users.

## Background

Care coordination is a central component of the current approach to achieving effective, efficient, high-quality care in the United States [1–3]. Effective care coordination is an increasing healthcare system priority, particularly in light of new Accountable Care Organizations policies that require health organizations to assume responsibility for patients across providers (e.g., physicians, nurse practitioners, and other clinicians) and settings (e.g., hospitals, clinics, and nursing homes) over time [4]. To improve coordination, some healthcare organizations have implemented a non-clinical boundary spanning position called the patient navigator to decrease fragmentation in the continuum of care [5, 6] and improve medical and administrative outcomes.

Patient navigation is a patient support intervention delivered by individuals without specific health care training who interact with patients in a peer-level capacity to facilitate health care delivery, patient-provider communication, and patient understanding of care issues [5]. The specific tasks of navigators may vary by program. Navigators usually assist patients with scheduling appointments and arranging transportation. They can also help with health insurance issues, public assistance, and other family or social problems [5, 7–9]. Patient navigation has been promoted most visibly by the National Cancer Institute, the American Cancer Society, and the Center for Medicare and Medicaid Services as an approach to improve patient engagement with the healthcare system and to address the needs of medically underserved cancer patients [6, 10–12].

Table 1 provides the common outcomes from patient navigation studies. Most published patient navigation research has focused on cancer screening and care in the primary care setting [6, 10, 13]. Navigation for cancer care is usually episode-based, and studies have examined screening time to intervention and treatment [10]. These studies demonstrated that patients receiving navigation had more timely screening and follow-through with diagnostic tests, as well as improved adherence and better engagement with the healthcare system [6, 10]. With a few exceptions [14–16], most studies found statistically significant differences between outcomes pre-navigation and post-navigation. These findings collectively suggest that patient navigation programs may be beneficial for achieving improved utilization and patient outcomes. However, the evidence supporting the efficacy of navigation for a broader range of chronic conditions remains limited, especially regarding its effect on medical and administrative outcomes [17, 18].

**Table 1:** Performance outcomes studied in the patient navigation literature at large - all conditions

Category	Performance Outcome	Authors
Adherence and Compliance to Clinical Practice Guidelines	Increased adherence to screening guidelines	[48, 49]
	Increased completion of screening (e.g., at first visit or follow-up)	[16, 50–53]
	Improved adherence to follow-up care	[15, 54, 55]
	Increased compliance with medication regimens	[29]
	Improved tracking of disease stage at diagnosis	[56]
Healthcare Utilization	Increased counseling participation	[14]
	Increased enrollment in pharmacy assistance programs	[29]
	Decreased no-show rate or “broken” appointments)	[51]
Efficiency	Improved timeliness (e.g., between referral and visit, to diagnostic resolution)	[14–16, 34, 55, 57, 58]
Patient Outcomes	Decreased scores on mental health screening instruments (anxiety, depression)	[57, 59]
	Increased patient satisfaction	[57]
	Increased desire for medical information	[59]
	Increased emotional and social quality of life	[59]
	Increased self-efficacy to cope with disease	[59]
	Improved physician-patient relationship	[59]
	Increased healthy birth outcomes in gestational diabetes cases	[29]
	Increased survival/decreased mortality	[56]
	Decreased A1C	[29, 60]

Patient navigation may be particularly helpful in diabetes care [19]. Successful diabetes control requires patients to carry out several self-management activities while working closely with medical providers on an ongoing basis. However, the significant shortage of diabetes care providers limits access to care and compromises adherence to clinical practice recommendations [20]. A large proportion of patients is subsequently at higher risk for poor disease control, complications, and frequent emergency department (ED) visits [21–23]. Patient navigators may help to bridge the gap between patients’ needs and clinic resources. Their efforts could lead to improvement in patient outcomes, especially for patients who are high-risk due to barriers to engagement with the healthcare system [24, 25] and who suffer from multiple complex diseases [21, 26–28]. Only two studies have examined specifically diabetes care “navigators” [29, 30]. The former did not evaluate the presence of differences in outcomes pre- versus post-navigation. Rather, it examined the percentage of navigated patients that experienced improved outcomes. The latter was a prospective interventional cohort study without a reference group involving patients with and at risk for type 2 diabetes from regular primary care practices. In contrast, our study compared patients in a navigation program with a matched reference group, which allowed us to better isolate the impact of the patient navigator intervention on observed outcomes.

To assess the effect of patient navigation on the medical and administrative outcome and process measures among patients with diabetes, we studied a navigation program at the outpatient diabetes clinic at Boston Medical Center, an urban safety-net hospital in Boston, Massachusetts.

## Methods

### Aims

The primary aims of the program were to improve glycemic control, increase patient engagement with the healthcare system, and improve the efficiency of care.

### Study design

Our study was a retrospective cohort study with pre- and post-intervention periods. The intervention group comprised individuals who participated in the patient navigation program; the reference group comprised patients who were eligible but did not participate in the program. The study was approved by the Institutional Review Board (IRB) at Boston Medical Center, and an appropriate patient consent process was followed based on the IRB requirements.

### Setting

The Endocrine, Diabetes, and Obesity Clinic at Boston Medical Center serves approximately 9000 predominantly minority, diabetes patients annually. On January 31, 2012, the clinic implemented a navigation program for high-risk patients.

### Participants

All patients enrolled in the diabetes clinic with A1C ≥ 8.5% and at least one appointment no-show in the past year were eligible for the navigation program. In addition, patients who did not meet the criteria were eligible if their health care provider requested the service.

Patients enrolled in the navigation program on a rolling basis starting on January 31, 2012. The original intent was to support patients for 180 days and then discharge them, but many remained enrolled for longer upon their request. For purposes of this study, January 31, 2014, was considered the last day of the program for those patients who were still enrolled on that day.

**Fig. 1:**
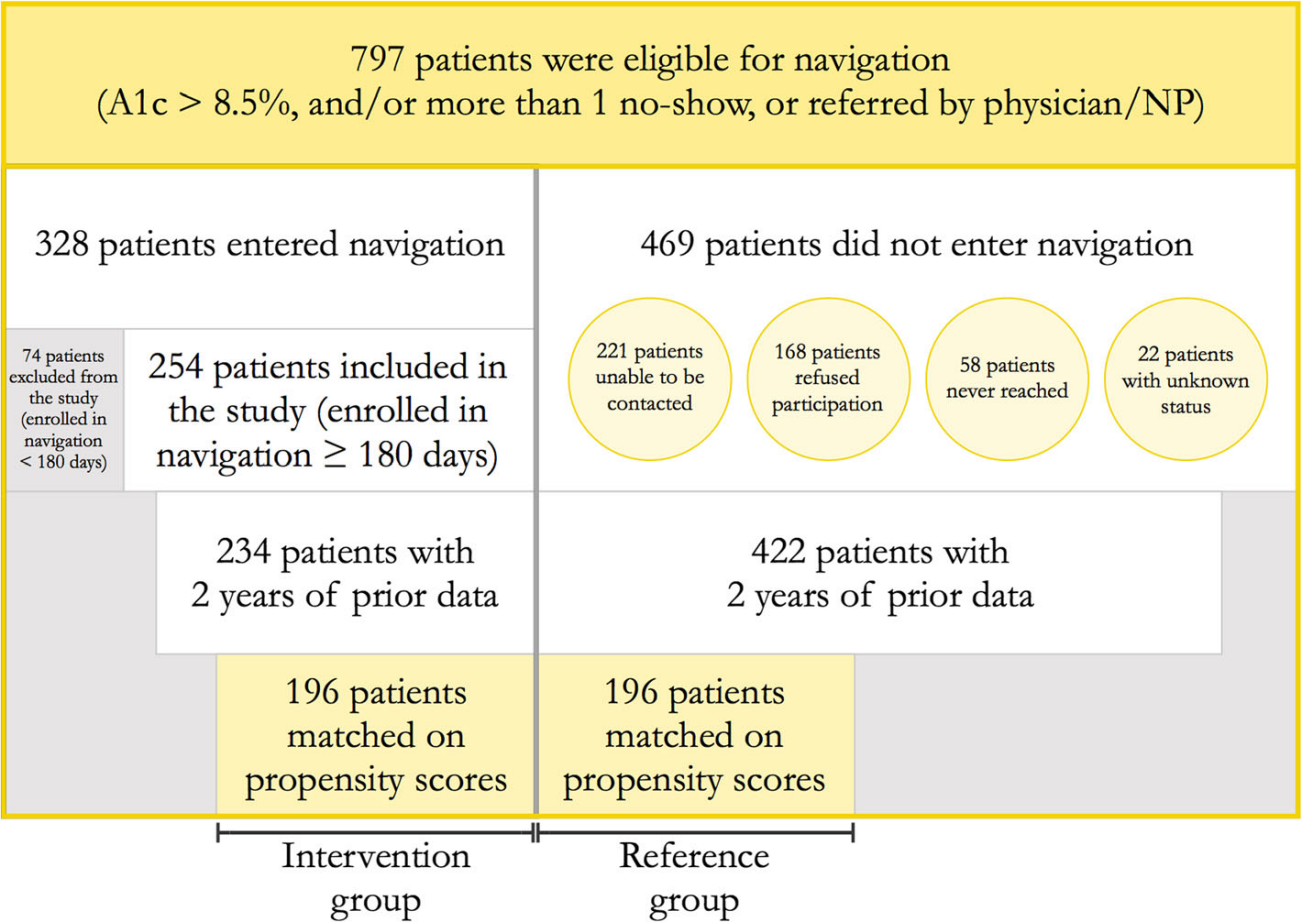
Recruitment of patients into the patient navigation program and the study

Patient selection for navigation and the study is illustrated in Fig. 1. Of the 797 eligible patients, 328 patients entered the program. Seventy-four of these patients dropped out in less than the intended 180 days and therefore were excluded from the analysis. The remaining 469 eligible patients did not enter the program because either they could not be contacted due to incorrect contact information or they declined navigation. Twenty patients who entered the program and 47 patients who did not have any relevant records in the electronic medical system throughout the baseline period and therefore had to be excluded from the study.

### Intervention

Two non-clinical navigators were recruited through job postings at Boston Medical Center and were selected based on their interpersonal and communication skills and experience in working with diverse populations on health-related outcomes. They had no previous specific education in diabetes care. This approach to selecting non-clinical navigators is similar to that used in other patient navigator programs [31]. Navigators received initial training, which included basic education on several topics: diabetes pathophysiology and treatment, provision of counseling and emotional support for diabetes self-management, care coordination, and cultural competency. They both also had training in procedures for patient navigation as part of a larger group of patient navigators in the hospital. The navigators were directly supervised by a nurse practitioner who specialized in diabetes care. Throughout the intervention, navigators were encouraged to participate in continuing education opportunities with the Diabetes Center staff and Boston Medical Center patient navigators from other specialties (e.g., cancer patient navigators). Navigator salaries were supported through research grants for the duration of the program. At the beginning of the intervention, the patient navigators were trained in Human Subjects Protection via the training provided by the hospital’s Office of Human Research Affairs.

To support diabetes-specific care goals, navigators worked with patients to identify and address barriers to care. Their responsibilities were to:

- Establish a personal connection with the patient
- Provide psychosocial support and basic clinical information related to diabetes self-management
- Educate patients on how to access needed services within the hospital system
- Remind patients to adhere to their medical and dietary regimen
- Identify and address logistical and personal barriers to self-management through arranging transportation, housing, social services, translator services and other support services
- Contact and schedule transportation, housing, social services, translator services and other support services when needed.
- Provide a reminder call to patients at least one day prior to a diabetes clinic appointment and periodically according to the navigator’s discretion
- Contact a patient who missed an appointment, i.e., “no shows”, within a week of the missed appointment and reschedule the appointment for the next available slot
- Enable care coordination between specialists and primary care physicians through informing clinicians of urgent or complex issues that could not be conveyed through the electronic medical record.
- As time allows, accompany patients to other diabetes-related appointments outside of the diabetes clinic, such as to Ophthalmology

Navigators related to patients on a peer level and interacted with them by phone and in person at the medical center. The number of contacts per patient was determined by each patient’s needs. One navigator spoke English only; the other spoke English and Spanish. The navigators were given a list of patients who met the study criteria each week. Patients were referred to the navigators several weeks before their next scheduled visit to the clinic and were assigned to each navigator based upon the available time in the navigators’ schedules. Hispanic/Latino patients were preferentially assigned to the navigator who was fluent in Spanish.

To ensure the fidelity of each interaction and the study, the patient navigators used a detailed navigator template form within the patient’s electronic medical record to support each interaction with a patient. The navigator template form was based on cancer care navigators in the hospital, but was adapted to fit the needs of the diabetes patient navigators. The fields on the template form included:

- Estimated time spent on interaction
- Incoming call
- Reminder call
- Appointment scheduling/rescheduling/canceling call
- Transportation barrier interaction
- Check-in interaction
- Housing barrier interaction
- Insurance barrier interaction
- Financial barrier interaction
- Literacy/education barrier interaction
- Childcare barrier interaction
- Social support interaction
- Language/interpreter barrier interaction
- Prescription barrier interaction
- Follow-up interaction
- Referral activity
- Accompanied patient to appointment

These fields were created for the navigator to easily record the issue or barrier the patient faced. There was also a notes section for the navigators to provide additional detail for each interaction. Each navigator was tasked with calling the patient or contacting the patient in person and retrieving data for these fields several weeks before their next scheduled visit to the clinic. Depending on the responses, the navigator was tasked with following-up with the appropriate support service and confirming the results of this follow-up with the patient. The navigator was also tasked with entering this follow-up into the navigator notes field for the patient. Each patient interaction (or interaction on behalf of the patient) was a separate note entry. Most interactions were completed prior to the patient’s appointment to ensure their appointment adherence; however, there were also instances of barriers to care that remained after the appointment (e.g., a prescription barrier interaction or referral activity) that the patient navigator may have needed to resolve after the appointment.

One of the researchers regularly met with the navigators to randomly review the patient notes to ensure consistency in implementation and to identify any challenges in reaching patients or recording their interactions.

### Measured outcomes:

1. Primary medical outcome: levels of A1C;
2. Secondary medical outcomes: levels of low-density lipoprotein (LDL) cholesterol, triglycerides, and random urine microalbumin;
3. Number of encounters: number of scheduled appointments, number of visits to the diabetes clinic, number of visits to the ED, number of inpatient stays; and
4. Scheduled appointment outcomes: percentage of arrivals, cancellations, and no-shows to scheduled appointments.

### Data sources

Data were obtained through the Boston Medical Center electronic medical record system and the hospital laboratory system.

### Statistical methods

Observational studies, like ours, may suffer from an imbalance between the intervention and reference groups in crucial covariates that may be associated with the studied outcomes. Therefore, we first applied the technique of propensity score matching to reduce selection bias and to strengthen the causal argument for the effect of patient navigation on the outcomes of interest [32]. The technique uses the observed baseline characteristics to estimate a propensity score (i.e., the probability of receiving the intervention) for each individual included in the study. Subsequently, it selects a subset of individuals from the study sample such that the propensity score distribution across the intervention group is equivalent to the propensity score distribution across the reference group. We estimated propensity scores using all available demographic information and baseline outcome levels. Those were calculated as person-level averages over the pre-intervention period from January 31, 2010, to January 30, 2012. If a patient had baseline records for some, but not all outcomes, we imputed the grand mean of the variable as the baseline value solely for purposes of the propensity scores estimation. We performed many iterations to find the most appropriate matching strategy to achieve a balance of covariates in terms of the minimum standardized differences while preserving most of the sample size.

We estimated the effect of patient navigation using difference-in-differences approach on the matched sample [33]. The difference-in-differences approach allows control for background changes in outcomes that occur with time and not due to the intervention. Medical outcomes were analyzed using an auto-regressive model with the normal distribution and identity link; numbers of encounters were analyzed using an auto-regressive model with the negative-binomial distribution and a log link; scheduled appointment outcomes were analyzed using a log-binomial model for a repeated binary outcome.

In sensitivity analyses, we used three different specifications of the pre- and post-intervention periods to assess the robustness of our results. First, for individuals in the intervention group we considered the time between January 31, 2010, and the patient-specific day before entering the program as the pre-intervention period, and the time spent in the program as the post-intervention periods. Such post-intervention period specification would capture the effect of the navigation program on currently enrolled patients. Second, for individuals in the intervention group we used the same pre-intervention period as in the former case, but we considered the time between entering the program and January 31, 2014, as the post-intervention period. Because most patients left the program well before January 31, 2014, we argue that such specification would capture whether the effects tended to remain or to fade away after quitting the program. Third, for individuals in the intervention group we considered the time in the program as the post-intervention period, and the same period shifted two years to the past as the pre-intervention period. We assumed that such specification would control for seasonal trends (e.g., fewer visits to the clinic during winter). For the reference group, we used January 31, 2010, to January 30, 2012, as the pre-intervention period, and January 31, 2012, to January 31, 2014, as the post-intervention period in all three specifications.

The statistical software R, version 3.2.2, was used for propensity score matching and for generating figures. Analyses were performed using SAS software, version 9.4 (SAS Institute Inc., Cary, North Carolina).

## Results

Prior to matching patients from the reference group to patients in the intervention group based on the estimated propensity scores, there were significant differences in demographics as well as in baseline outcome levels between the two groups. They differed in Charlson Comorbidity Index (*p* = .033), ethnicity (*p* < .001), education (*p* = .012), employment status (*p* = .023), baseline urine microalbumin level (*p* = .045), baseline number of scheduled appointments (*p* = .004), baseline number of visits to the clinic (*p* < .001), baseline percentage of arrivals (*p* = .002), and baseline percentage of no-shows (*p* = .001). Standardized differences were often greater than 10%. Propensity score matching reduced the sample size to 392 individuals (196 in each group), but improved the balance of covariates across the two groups substantially (Additional file 1: Figure A1). All standardized differences were less than 10% after matching. Table 2 shows the demographics and baseline outcome values in the original and matched samples.

**Table 2:** Demographics and baseline characteristics

	Original sample				Matched sample		
Characteristic	Intervention group (*n* = 234)	Reference group (*n* = 422)	*p*-value	Standardized difference (%)	Intervention group (*n* = 196)	Reference group (*n* = 196)	Standardized difference (%)
Age (± *sd*)	56.3 (± 13.6)	55.7 (± 13.6)	.61	4.2	56.0 (± 14.1)	56.0 (± 13.7)	0.3
Charlson Comorbidity Index (± *sd*)	2.8 (± 2.3)	2.4 (± 1.9)	.033*	17.9^a^	2.7 (± 2.1)	2.7 (± 2.0)	−1.5
Female	104 (44.4%)	206 (48.8%)	.32	−8.8	85 (43.4%)	91 (46.4%)	−6.2
Homeless	12 (5.1%)	23 (5.5%)	.99	−1.4	11 (5.6%)	9 (4.6%)	4.6
Ethnicity							
Black/African American	103 (44.0%)	259 (61.4%)	<.001***	−35.3^a^	98 (50.0%)	106 (54.1%)	−8.2
Hispanic/Latino	105 (44.9%)	66 (15.6%)		67.1^a^	73 (37.2%)	64 (32.7%)	9.6
White	19 (8.1%)	66 (15.6%)		−23.4^a^	19 (9.7%)	19 (9.7%)	0.0
Other	7 (3.0%)	31 (7.3%)		−19.8^a^	6 (3.1%)	7 (3.6%)	−2.8
Education							
Did not attend school	19 (8.1%)	15 (3.6%)	.012*	19.6^a^	17 (8.7%)	13 (6.6%)	7.7
8th grade or less	23 (9.8%)	21 (5.0%)		18.6^a^	15 (7.7%)	12 (6.1%)	6.0
Some high school	68 (29.1%)	122 (28.9%)		0.3	52 (26.5%)	59 (30.1%)	−7.9
High school or GED	81 (34.6%)	158 (37.4%)		−5.9	73 (37.2%)	73 (37.2%)	0.0
Some college/Voc./Tech.	17 (7.3%)	38 (9.0%)		−6.4	15 (7.7%)	16 (8.2%)	−1.9
College/Postgraduate	13 (5.6%)	44 (10.4%)		−18.0^a^	12 (6.1%)	12 (6.1%)	0.0
Other	13 (5.6%)	24 (5.7%)		−0.6	12 (6.1%)	11 (5.6%)	2.2
Employment status							
Full-time	19 (8.1%)	70 (16.6%)	.023*	−26.0^a^	18 (9.2%)	20 (10.2%)	−3.4
Part-time	7 (3.0%)	23 (5.5%)		−12.3^a^	6 (3.1%)	9 (4.6%)	−8.0
Unemployed	101 (43.2%)	150 (35.5%)		15.6^a^	83 (42.3%)	79 (40.3%)	4.1
Disabled	55 (23.5%)	93 (22.0%)		3.5	43 (21.9%)	48 (24.5%)	−6.0
Retired	36 (15.4%)	55 (13.0%)		6.7	31 (15.8%)	27 (13.8%)	5.8
Other	16 (6.8%)	31 (7.3%)		−2.0	15 (7.7%)	13 (6.6%)	4.0
Health insurance							
Commercial/Private	24 (10.3%)	72 (17.1%)	.18	−19.9^a^	22 (11.2%)	18 (9.2%)	6.7
Medicaid	103 (44.0%)	171 (40.5%)		7.1	84 (42.9%)	89 (45.4%)	−5.1
Medicare	84 (35.9%)	140 (33.2%)		5.7	70 (35.7%)	72 (36.7%)	−2.1
Charity	16 (6.8%)	23 (5.5%)		5.8	13 (6.6%)	11 (5.6%)	4.3
Other	7 (3.0%)	16 (3.8%)		−4.4	7 (3.6%)	6 (3.1%)	2.8
Marital status							
Single	120 (51.3%)	231 (54.7%)	.81	−6.9	103 (52.6%)	102 (52.0%)	1.0
Married	65 (27.8%)	113 (26.8%)		2.2	50 (25.5%)	58 (29.6%)	−9.1
Separated	18 (7.7%)	24 (5.7%)		8.0	14 (7.1%)	12 (6.1%)	4.1
Divorced	19 (8.1%)	36 (8.5%)		−1.5	17 (8.7%)	16 (8.2%)	1.8
Widowed	12 (5.1%)	18 (4.3%)		4.1	12 (6.1%)	8 (4.1%)	9.3
Baseline^b^ medical outcomes							
A1C (%)	9.6 (± 1.9)	9.6 (± 2.0)	.98	−0.2	9.7 (± 2.0)	9.6 (± 2.1)	2.7
LDL cholesterol (mmol/l)	99.5 (± 32.8)	103.2 (± 33.6)	.21	−11.0^a^	100.3 (± 33.9)	99.8 (± 34.0)	1.5
Triglycerides (mg/dl)	182.1 (± 144.8)	174.5 (± 153.5)	.56	5.1	179.3 (± 149.4)	172.9 (± 108.4)	4.9
Urine microalbumin (mg)	217.4 (± 520.4)	135.5 (± 396.5)	.045*	17.7^a^	161.3 (± 378.6)	179.2 (± 513.9)	−4.0
Baseline^b^ encounters							
Appointments (per year)	3.9 (± 3.6)	3.1 (± 3.6)	.004**	23.5^a^	3.6 (± 3.5)	3.7 (± 4.2)	−4.4
Clinic visits (per year)	17.0 (± 13.5)	13.4 (± 12.8)	<.001***	26.9^a^	15.8 (± 12.6)	15.7 (± 14.1)	0.1
ER visits (per year)	1.0 (± 1.6)	0.9 (± 1.6)	.67	3.5	1.1 (± 1.8)	0.9 (± 1.6)	8.1
Inpatient stays (per year)	0.5 (± 0.9)	0.5 (± 1.0)	.67	3.4	0.4 (± 0.8)	0.5 (± 0.8)	−4.5
Baseline^b^ appointment outcomes							
Arrival (%)	50.7 (± 25.6)	42.7 (± 30.2)	.002**	28.4^a^	48.5 (± 26.6)	47.9 (± 29.9)	2.5
Cancellation (%)	19.7 (± 20.1)	18.7 (± 20.6)	.60	5.0	20.0 (± 20.5)	19.8 (± 22.2)	0.7
No-show (%)	29.7 (± 25.9)	38.6 (± 33.6)	.001**	−29.9^a^	31.5 (± 27.4)	32.3 (± 32.1)	−2.8

*Significant at *p* ≤ .05; ** significant at *p* ≤ .01; *** significant at *p* ≤ .001

^a^Absolute value of mean standardized difference above 10%

^b^Baseline characteristics were calculated as person-level averages over a 2-year period (January 31, 2010 – January 30, 2012) before the patient navigation program initiation

Table 3 presents information on how navigators spent their time. These data were extracted from the navigator template form in the medical record for each participant. The navigators spent a median time of 186 min navigating each patient, including 129 min directly with the patient and a median of 42 min coordinating various activities for the patient. Appointment reminder calls were the most frequent navigator activity (median: 5 calls) followed by calls to follow up with the patient after an appointment (median: 3 calls).

**Table 3:** Descriptive statistics of the navigators’ activity

	Median	IQR
Total time spent navigating a patient (min)	186	99–323
Interacting directly with a patient (min)	129	72–225
Coordinating various activities for a patient (min)	42	14–84
Number of direct interactions between navigator and patient	1	0–2
Number of incoming call interactions	0	0–1
Number of contacts of patient to check-in	0	0–1
Number of appointment reminder calls	5	2–7
Number of contacts of patient to follow-up	3	1–4
Number of interactions to schedule/reschedule/cancel appointments	0	0–0
Number of other phone calls	0	0–1

Estimated means of the studied outcomes in each group during the pre- and post-intervention periods are reported in Table 4. We describe the effects of the navigation program as the difference-in-differences of the estimated means in both study groups between the pre- and post-intervention periods, and we reported the interaction term *p*-value to indicate statistical significance. Detailed results of the estimated models are presented in Additional file 1.

**Table 4:** Estimated means of medical and administrative outcomes

Outcome	Intervention group (n = 196)		Reference group (n = 196)			
Period	Mean	95% CI	Mean	95% CI	DD^a^	*p-*value^b^
A1C (%)						
Pre-intervention	9.9	(9.7, 10.2)	9.4	(9.2, 9.7)	−1.1	<.001***
Post-intervention	9.3	(9.1, 9.6)	9.9	(9.6, 10.2)		
LDL cholesterol (mmol/l)						
Pre-intervention	101.9	(97.0, 106.8)	101.9	(96.7, 107.0)	−0.8	.81
Post-intervention	102.3	(96.8, 107.8)	103	(97.8, 108.3)		
Triglycerides (mg/dl)						
Pre-intervention	185.5	(161.1, 209.9)	178.1	(158.0, 198.2)	−14.8	.28
Post-intervention	193.2	(171.9, 214.4)	200.5	(171.6, 229.4)		
Urine microalbumin (mg)						
Pre-intervention	152.2	(94.0, 210.4)	169.9	(100.4, 239.5)	−5.5	.87
Post-intervention	143.3	(81.7, 204.9)	166.6	(116.8, 216.3)		
	*N*	95% CI	*N*	95% CI	DD^a^	*p-*value^b^
Appointments (per year)						
Pre-intervention	3.8	(3.4, 4.3)	3.7	(3.2, 4.4)	+5.3	<.001***
Post-intervention	10.6	(9.7, 11.6)	5.2	(4.7, 5.8)		
Clinic visits (per year)						
Pre-intervention	16.2	(14.6, 18.0)	15.8	(14.0, 17.9)	+6.4	<.001***
Post-intervention	22.1	(20.0, 24.5)	15.3	(13.6, 17.2)		
ED visits (per year)						
Pre-intervention	1.1	(0.9, 1.4)	1.0	(0.7, 1.2)	+0.3	.13
Post-intervention	1.4	(1.1, 1.7)	0.9	(0.7, 1.1)		
Inpatient stays (per year)						
Pre-intervention	0.5	(0.4, 0.6)	0.5	(0.4, 0.6)	+0.1	.49
Post-intervention	0.5	(0.4, 0.6)	0.4	(0.3, 0.6)		
	%	95% CI	%	95% CI	DD^a^	*p-*value^b^
Arrival (%)						
Pre-intervention	53.2	(49.4, 56.9)	55.6	(51.6, 59.4)	+7.4	.009**
Post-intervention	53.2	(49.7, 56.7)	48.2	(44.7, 51.8)		
Cancellation (%)						
Pre-intervention	22.2	(19.6, 25.0)	21.5	(18.2, 25.2)	+2.2	.39
Post-intervention	26.6	(23.9, 29.4)	23.7	(20.5, 27.2)		
No-show (%)						
Pre-intervention	24.6	(21.6, 28.0)	22.8	(19.8, 26.0)	−9.8	<.001***
Post-intervention	20.2	(17.6, 23.0)	28.2	(25.0, 31.7)		

*Significant at *p* ≤ .05; **significant at *p* ≤ .01; *** significant at *p* ≤ .001

^a^DD = Difference-in-differences

^b^Each *p*-value corresponds to the interaction term of the estimated auto-regressive model for the particular outcome

### Medical outcomes

The mean A1C level among patients in the intervention group improved from 9.9% to 9.3% (difference of −0.6 percentage points), while the average A1C level among patients in the reference group worsened from 9.4% to 9.9% (difference of +0.5 percentage points). The overall effect of patient navigation on A1C is the difference of these differences, i.e. a significant 1.1 percentage point (*p* < .001) decrease in A1C. There was no significant effect on other medical outcomes studied. The results were robust to changes in the pre- and post-intervention period specification.

Additional file 1 Figure A2 shows changes in the distribution of each medical outcome over the course of the study. For example, the top left graph in Additional file 1: Figure A2 represents the change of the A1C distribution between the pre-intervention and post-intervention periods among navigated patients. The graph suggests that there were fewer patients with A1C in the 12–16% range and more patients with A1C in the 8–10% range in the post-intervention period compared to the pre-intervention period. Moreover, the top right graph in Additional file 1: Figure A2 shows an overall shift to the right of the A1C distribution among patients in the reference group, demonstrating that the A1C level in patients in the reference group increased

### Number of encounters

The mean number of scheduled appointments increased substantially in the intervention group, although it slightly increased in the reference group as well. The net effect was an increase by 5.3 scheduled appointments annually (*p* < .001). The mean number of attended visits to the clinic per year increased from 16.2 to 22.1 in the intervention group, while the number slightly decreased from 15.8 to 15.3 in the reference group. Thus, the net effect was an increase of 6.4 attended visits to the clinic annually (*p* < .001).

**Fig. 2:**
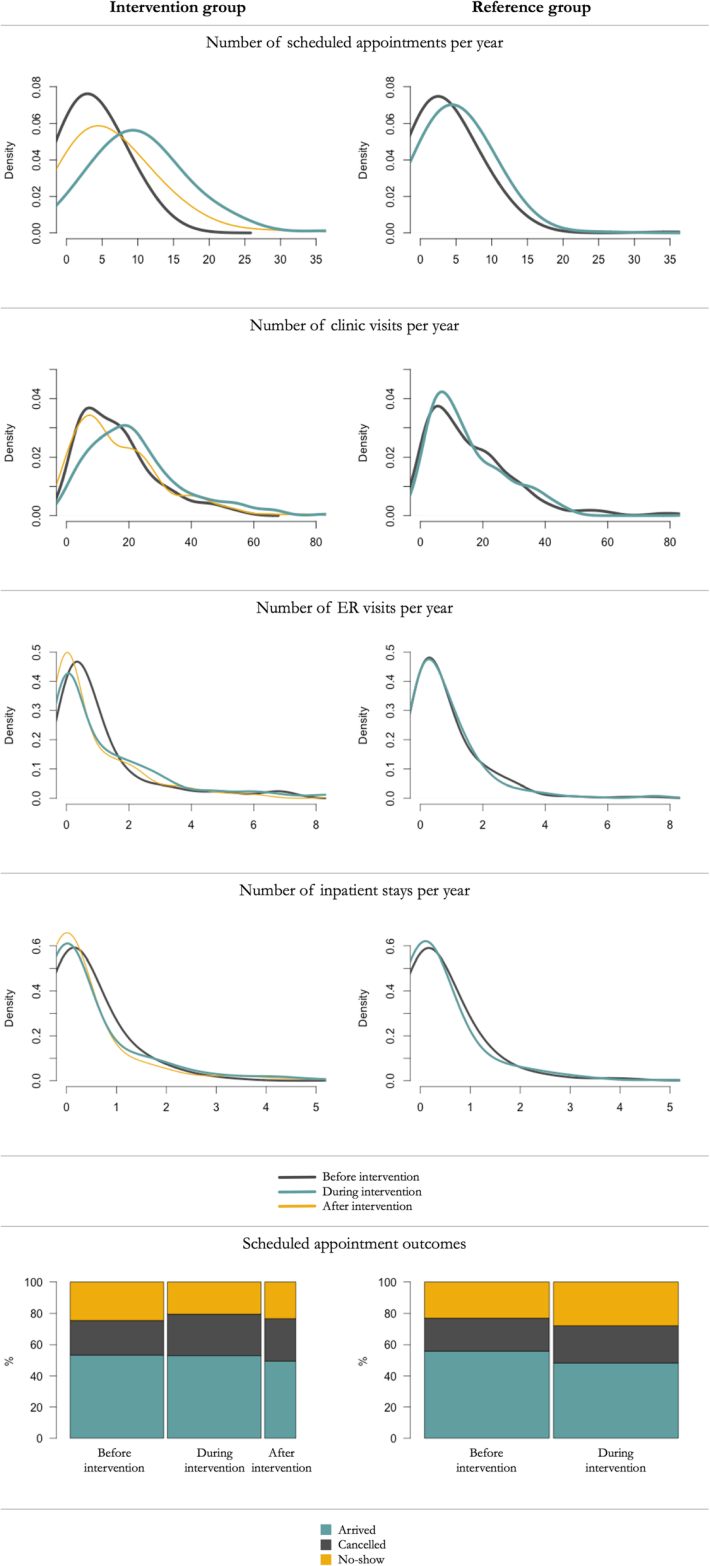
Comparison of changes in the distribution of administrative outcomes in each study group over the course of the study

Figure 2 shows changes in the distribution of administrative outcomes over the course of the study. For example, the top left graph illustrates the substantial increase in scheduled appointments among patients who were currently enrolled in the navigation program. It also suggests that the effect faded away after the patients left the program. This finding is also supported by parameter estimates in the sensitivity analysis by alteration of the pre- and post-intervention period specification. While the effect was statistically significant for both outcomes under all three pre- and post-intervention period specifications, the magnitude of the effect was lower under the specification using the time since enrollment until January 31, 2014, as the post-intervention period, despite that many patients left the program before that day.

Patient navigation was not significantly associated with a change in the number of ED visits or the number of inpatient stays. The results were robust to changes in the pre- and post-intervention period specification for inpatient stays; however, there was an increase by 0.4 ED visits per year (*p* = .025) under the specification controlling for seasonal variation.

### Scheduled appointment outcomes

The percentage of arrivals to scheduled appointments remained constant at 53.2% in the intervention group; however, it decreased from 55.6% to 48.2% in the reference group leading to a net effect of 7.4 percentage points (*p* = .009) increase in arrivals. The percentage of appointment cancellations rose in both groups, and the overall effect was not statistically significant (*p* = .39). Finally, the percentage of no-shows to scheduled appointments decreased from 24.6% to 20.2% in the intervention group and increased from 22.8% to 28.2% in the reference group. Thus, the overall effect was a decrease in no-shows by 9.8 percentage points (*p* < .001). The results were relatively robust to changes in the pre- and post-intervention period specification; however, Fig. 3 suggests a slight increase in no-shows among patients who left the program.

## Discussion

Our study demonstrates that a patient navigation program for diabetes, using non-clinical peer navigators, can improve medical and administrative outcomes among patients with diabetes. Patients participating in our navigation program were predominantly minority, urban, and of low socioeconomic status [10, 34]. These groups generally have the greatest barriers to engagement with the healthcare system and the worst health outcomes [12, 21]. On average, patients who participated in the navigation program showed a clinically significant reduction in A1C level, scheduled more appointments, had more visits to the clinic and were less likely to miss scheduled appointments compared to eligible patients who did not participate in the program.

Improved glycemic control was a central goal of the patient navigation program, and the 1% improvement in A1C we observed among navigation participants is an important finding. In seminal studies of glycemic control and long-term complications of diabetes, each 1% drop in A1C was associated with a 40% decrease in the risk of developing eye, kidney, and nerve complications [35, 36], as well as a reduction in the progression of existing complications. Ongoing engagement with navigators may be needed to sustain this improvement in glycemic control. While the mean A1C did not reach ideal treatment targets, the 1% A1C reduction among patients with multiple socioeconomic barriers to optimal diabetes control suggests that a patient navigation program can result in clinically significant improvement in an important parameter of diabetes management.

The navigation program did not demonstrate improvement in LDL cholesterol, triglycerides, or urine microalbumin. Lipid control is complicated by the fact that nonadherence to treatment is very common [37] and often goes unmentioned by patients. Urine microalbumin level depends on both glycemic control and kidney function, which can itself be affected by blood pressure control – in part a function of medication adherence. While navigators frequently checked in with patients about problems they were having with self-management and medication refills, assessing adherence to specific medications was not an explicit navigator responsibility. One improvement to the patient navigation program would be to build in a formalized brief assessment of medication adherence. Patients may be more willing to disclose nonadherence to a peer navigator than to a physician. Several self-report adherence scales have been validated for diabetes, such as the Medication Adherence Questionnaire (MAQ), also known as the 4-item Morisky Medication Adherence Scale (MMAS-4). The MAQ is quick to administer and score; navigators could readily complete the MAQ with patients and provide that information to clinicians. Such an approach could increase the effectiveness of navigators in addressing the range of cardiovascular risk factors that impact the risk of diabetes complications.

We observed greater engagement in the diabetes clinic among the program participants as evidenced by more scheduled appointments and fewer missed appointments. Navigators spent 25% of their time on coordination activities on behalf of patients, and their most frequent activities were related to appointment scheduling and adherence. Because navigators’ work to arrange clinic visits was highly proactive and directly linked to their knowledge of patients’ clinical needs, the appointment-related work they did was qualitatively different and more comprehensive than that provided by the usual clinic staff’s appointment reminder phone calls. For example, while a staff member would simply call and remind the patient of an appointment, a patient navigator was tasked with reminding the patient and asking if there were any barriers to the patient making the appointment and alleviating those barriers to the best of the patient navigator’s ability. Navigated patients had, on average, six more visits to the diabetes clinic and a 10% lower rate of no-shows as compared to the reference group. Missed appointments [24] and fewer clinic visits [38] are associated with worse glycemic control; increased clinic engagement is likely to have been an important factor in the improved A1C we observed. In addition to improving clinical parameters, this improvement in clinic engagement is likely to have a positive impact on clinic revenue and should be quantified in future work.

The aim of patient navigation programs and similar coordination programs has been to shift costly health care utilization through ED visits, and inpatient stays to more desirable utilization through scheduled appointments and non-emergency outpatient visits [39]. Although our study documented a beneficial impact of navigation on scheduled diabetes clinic visits, we found no evidence of a change in either the number of inpatient stays or the number of ED visits. Neither of these outcomes was specific to diabetes care. More research is required to understand better the impact of navigation on these outcomes, which should include a focus on the diabetes-specific use of non-routine services.

This is one of the few studies, of which we are aware, that examine how diabetes care is improved by patient navigation. Other studies of care coaches for diabetes patients report reductions in A1C similar to our findings [40–42]. Patient navigators are similar to care coaches in that they can play a wide range of roles in the healthcare system and interact with patients via phone [5]. However, their roles differ in important ways. Patient navigators often have the opportunity for face-to-face relationships with patients and they have direct channels of communication with clinical and administrative staff [5, 6]. Their role may be more long-term and more involved than the traditional care coaches’ role [8, 43, 44]. Peer coaches are individuals who share the index condition of the patient and draw on their experiential knowledge to guide their mentees [45]. The patient navigators in this study were trained to relate to patients as peers rather than as health care professionals, but they did not have diabetes.

Our patient navigators had an interest in healthcare but no personal experience with or formal educational background about diabetes, which may be a more replicable model. Despite navigators’ lack of personal experience with diabetes, patients who interacted with them had better outcomes than those who did not. Anecdotally, we found that many patients did not want to leave the program when their 180 days were completed. It may be that patients formed trusting and productive relationships with their assigned navigator, and that effective patient-navigator communication allowed patients a better understanding of and buy-in to the treatment plan.

Our study has several limitations. First, it was a clinical program offered as part of the regular suite of services, not a randomized controlled trial. To mitigate this problem, we used propensity score matching to balance the study groups on all observed characteristics and baseline outcome levels [32]. Subsequently, by using the difference-in-differences approach on the matched samples, we were able to isolate the effect of patient navigation on several outcomes, and therefore we make a strong case for causal inference [33, 46]. Moreover, we performed a sensitivity analysis by varying the length of the pre- and post-intervention periods, which confirmed that our results were robust and lent credence to our findings. Second, we excluded individuals who participated in the program for less than 180 days. The program might have had some adverse effects causing certain individuals to discontinue their participation; we did not capture this data. Third, most of the patients in the reference group refused navigation or were not approachable; this may introduce variables that are not fully corrected for with propensity score matching. Fourth, the size of our program permitted only two navigators, and we could not determine to what extent the success of our program was influenced by their personal characteristics. To partially standardize the service, we provided both navigators with the tools they needed to complete their work effectively. These included replicable components: basic diabetes education and support training, the ability to book appointments, and ready access to clinicians. Finally, the program was in an urban non-profit hospital and may not be generalizable to other systems.

## Conclusions

Overall, our study provides encouraging evidence that patient navigation can improve glycemic control and health care utilization for patients with diabetes in a safety-net setting. The intervention could be a useful component of a broader program for managing chronic complex conditions. However, this patient navigation approach requires more continued rigorous evaluation to understand what features of it are most important to effectiveness, its replicability across other chronic disease states, and whether the program can be sustainable. Reviews have found that the effect of many chronic disease interventions is not sustained when contact from the intervention is finished [47]. Follow-up on the status of these patients in the future will help to determine whether a time-limited intervention by navigators can have a lasting effect.

## Additional file

Additional file 1: Complete results of the analysis (DOCX 392 kb)

## Abbreviations

A1C: Hemoglobin A1c
ED: Emergency Department
IRB: Institutional Review Board
LDL: Low-density lipoprotein
MAQ: Medication Adherence Questionnaire
MMAS-4: Morisky Medication Adherence Scale
