# Supplementary material for: Patient navigation to improve diabetes outpatient care at a safety-net hospital: a retrospective cohort study
Source: BMC Health Serv Res. 2017 Nov 21;17:759. doi: 10.1186/s12913-017-2700-7 (PMC5699176; doi:10.1186/s12913-017-2700-7)
Supplement: Additional file 1: — Complete results of the analysis (DOCX 392 kb) [file 12913_2017_2700_MOESM1_ESM.docx]

# Additional file 1

Figure A1: Propensity scores matching summary

| **** |
| --- |
| **** |

Figure A2: Comparison of changes in the distribution of medical outcomes in each study group over the course of the study


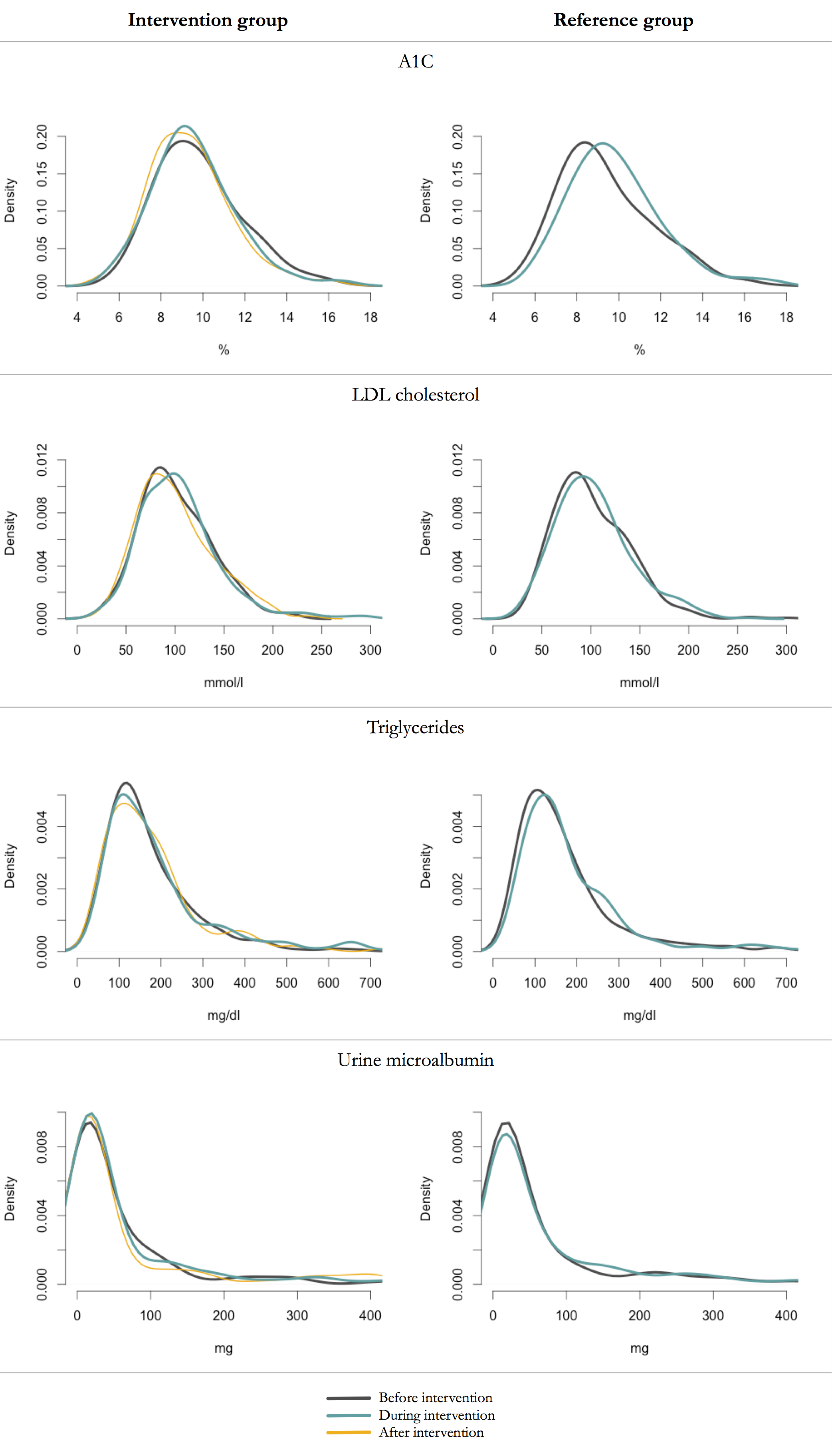


Table B1: A_1C_

|  | (1) | | | |  | (2) | | | |  | (3) | | | |
| --- | --- | --- | --- | --- | --- | --- | --- | --- | --- | --- | --- | --- | --- | --- |
|  | Est. | 95% CI | *p*-value | |  | Est. | 95% CI | *p*-value | |  | Est. | 95% CI | *p*-value | |
| Intercept | 9.44 | (9.15, 9.72) | <.001 | *** |  | 9.44 | (9.15, 9.72) | <.001 | *** |  | 9.44 | (9.15, 9.72) | <.001 | *** |
| Intervention group | 0.49 | (0.11, 0.87) | .012 | * |  | 0.48 | (0.10, 0.86) | .012 | * |  | 0.11 | (-0.33, 0.54) | .63 | . |
| Post period | 0.47 | (0.20, 0.75) | .001 | ** |  | 0.47 | (0.20, 0.75) | .001 | ** |  | 0.47 | (0.20, 0.75) | .001 | ** |
| Intervention group × Post period | -1.07 | (-1.43, -0.72) | <.001 | *** |  | -1.09 | (-1.44, -0.74) | <.001 | *** |  | -0.41 | (-0.82, -0.01) | .047 | * |

*Notes*: * significant at *p* ≤ .05; ** significant at *p* ≤ .01; *** significant at *p* ≤ .001.

Specification (1): pre-intervention period = from January 31, 2010, to the patient-specific day before entering the program; post-intervention period = the time spent in the navigation program.

Specification (2): pre-intervention period = from January 31, 2010, to the patient-specific day before entering the program; post-intervention period = from the patient-specific day of entering the program to January 31, 2014.

Specification (3): pre-intervention period = the time spent in the navigation program shifted two years to the past; post-intervention period = the time spent in the navigation program.

Table B2: LDL cholesterol

|  | (1) | | | |  | (2) | | | |  | (3) | | | |
| --- | --- | --- | --- | --- | --- | --- | --- | --- | --- | --- | --- | --- | --- | --- |
|  | Est. | 95% CI | *p*-value | |  | Est. | 95% CI | *p*-value | |  | Est. | 95% CI | *p*-value | |
| Intercept | 101.86 | (96.71, 107.01) | <.001 | *** |  | 101.86 | (96.71, 107.01) | <.001 | *** |  | 101.55 | (96.49, 106.62) | <.001 | *** |
| Intervention group | 0.04 | (-7.08, 7.17) | .99 | . |  | 0.01 | (-7.09, 7.12) | .99 | . |  | -0.42 | (-8.30, 7.46) | .92 | . |
| Post period | 1.16 | (-3.39, 5.71) | .62 | . |  | 1.16 | (-3.39, 5.71) | .62 | . |  | 1.77 | (-2.72, 6.27) | .44 | . |
| Intervention group × Post period | -0.78 | (-7.06, 5.51) | .81 | . |  | -1.62 | (-7.54, 4.30) | .59 | . |  | -0.03 | (-7.16, 7.10) | .99 | . |

*Notes*: * significant at *p* ≤ .05; ** significant at *p* ≤ .01; *** significant at *p* ≤ .001.

Specification (1): pre-intervention period = from January 31, 2010, to the patient-specific day before entering the program; post-intervention period = the time spent in the navigation program.

Specification (2): pre-intervention period = from January 31, 2010, to the patient-specific day before entering the program; post-intervention period = from the patient-specific day of entering the program to January 31, 2014.

Specification (3): pre-intervention period = the time spent in the navigation program shifted two years to the past; post-intervention period = the time spent in the navigation program.

Table B3: Triglycerides

|  | (1) | | | |  | (2) | | | |  | (3) | | | |
| --- | --- | --- | --- | --- | --- | --- | --- | --- | --- | --- | --- | --- | --- | --- |
|  | Est. | 95% CI | *p*-value | |  | Est. | 95% CI | *p*-value | |  | Est. | 95% CI | *p*-value | |
| Intercept | 178.05 | (157.95, 198.15) | <.001 | *** |  | 178.08 | (157.94, 198.22) | <.001 | *** |  | 175.51 | (156.52, 194.5) | <.001 | *** |
| Intervention group | 7.45 | (-24.18, 39.07) | .64 | . |  | 7.10 | (-24.62, 38.81) | .66 | . |  | -2.05 | (-38.35, 34.26) | .91 | . |
| Post period | 22.41 | (2.55, 42.28) | .027 | * |  | 22.58 | (2.67, 42.50) | .026 | * |  | 18.21 | (-1.55, 37.96) | .07 | . |
| Intervention group × Post period | -14.76 | (-41.29, 11.76) | .28 | . |  | -18.31 | (-43.92, 7.30) | .16 | . |  | W2.83 | (-28.25, 33.91) | .86 | . |

*Notes*: * significant at *p* ≤ .05; ** significant at *p* ≤ .01; *** significant at *p* ≤ .001.

Specification (1): pre-intervention period = from January 31, 2010, to the patient-specific day before entering the program; post-intervention period = the time spent in the navigation program.

Specification (2): pre-intervention period = from January 31, 2010, to the patient-specific day before entering the program; post-intervention period = from the patient-specific day of entering the program to January 31, 2014.

Specification (3): pre-intervention period = the time spent in the navigation program shifted two years to the past; post-intervention period = the time spent in the navigation program.

Table B4: Urine microalbumin

|  | (1) | | | |  | (2) | | | |  | (3) | | | |
| --- | --- | --- | --- | --- | --- | --- | --- | --- | --- | --- | --- | --- | --- | --- |
|  | Est. | 95% CI | *p*-value | |  | Est. | 95% CI | *p*-value | |  | Est. | 95% CI | *p*-value | |
| Intercept | 169.94 | (100.41, 239.46) | <.001 | *** |  | 169.36 | (99.56, 239.16) | <.001 | *** |  | 169.83 | (100.49, 239.16) | <.001 | *** |
| Intervention group | -17.73 | (-108.40, 72.95) | .70 | . |  | -12.92 | (-102.82, 76.98) | .78 | . |  | -16.59 | (-124.86, 91.67) | .76 | . |
| Post period | -3.37 | (-46.43, 39.68) | .88 | . |  | -1.56 | (-43.03, 39.92) | .94 | . |  | -3.47 | (-47.33, 40.38) | .88 | . |
| Intervention group × Post period | -5.54 | (-73.65, 62.57) | .87 | . |  | -4.65 | (-67.60, 58.29) | .88 | . |  | -16.08 | (-107.78, 75.63) | .73 | . |

*Notes*: * significant at *p* ≤ .05; ** significant at *p* ≤ .01; *** significant at *p* ≤ .001.

Specification (1): pre-intervention period = from January 31, 2010, to the patient-specific day before entering the program; post-intervention period = the time spent in the navigation program.

Specification (2): pre-intervention period = from January 31, 2010, to the patient-specific day before entering the program; post-intervention period = from the patient-specific day of entering the program to January 31, 2014.

Specification (3): pre-intervention period = the time spent in the navigation program shifted two years to the past; post-intervention period = the time spent in the navigation program.

Table C1: Number of appointments

|  | (1) | | | |  | (2) | | | |  | (3) | | | |
| --- | --- | --- | --- | --- | --- | --- | --- | --- | --- | --- | --- | --- | --- | --- |
|  | Est. | 95% CI | *p*-value | |  | Est. | 95% CI | *p*-value | |  | Est. | 95% CI | *p*-value | |
| Intercept | 1.31 | (1.16, 1.47) | <.001 | *** |  | 1.31 | (1.16, 1.47) | <.001 | *** |  | 1.31 | (1.16, 1.47) | <.001 | *** |
| Intervention group | 0.02 | (-0.18, 0.22) | .84 | . |  | 0.02 | (-0.18, 0.22) | .84 | . |  | -0.03 | (-0.25, 0.20) | .82 | . |
| Post period | 0.34 | (0.19, 0.49) | <.001 | *** |  | 0.34 | (0.19, 0.49) | <.001 | *** |  | 0.34 | (0.19, 0.49) | <.001 | *** |
| Intervention group × Post period | 0.68 | (0.48, 0.89) | <.001 | *** |  | 0.53 | (0.32, 0.74) | <.001 | *** |  | 0.73 | (0.50, 0.97) | <.001 | *** |

*Notes*: * significant at *p* ≤ .05; ** significant at *p* ≤ .01; *** significant at *p* ≤ .001.

Specification (1): pre-intervention period = from January 31, 2010, to the patient-specific day before entering the program; post-intervention period = the time spent in the navigation program.

Specification (2): pre-intervention period = from January 31, 2010, to the patient-specific day before entering the program; post-intervention period = from the patient-specific day of entering the program to January 31, 2014.

Specification (3): pre-intervention period = the time spent in the navigation program shifted two years to the past; post-intervention period = the time spent in the navigation program.

Table C2: Number of clinic visits

|  | (1) | | | |  | (2) | | | |  | (3) | | | |
| --- | --- | --- | --- | --- | --- | --- | --- | --- | --- | --- | --- | --- | --- | --- |
|  | Est. | 95% CI | *p*-value | |  | Est. | 95% CI | *p*-value | |  | Est. | 95% CI | *p*-value | |
| Intercept | 2.76 | (2.64, 2.88) | <.001 | *** |  | 2.76 | (2.64, 2.88) | <.001 | *** |  | 2.76 | (2.63, 2.88) | <.001 | *** |
| Intervention group | 0.03 | (-0.14, 0.19) | 0.75 | . |  | 0.03 | (-0.13, 0.19) | .74 | . |  | -0.04 | (-0.22, 0.14) | .67 | . |
| Post period | -0.03 | (-0.14, 0.07) | 0.56 | . |  | -0.03 | (-0.14, 0.08) | .58 | . |  | -0.03 | (-0.14, 0.08) | .58 | . |
| Intervention group × Post period | 0.34 | (0.20, 0.48) | <.001 | *** |  | 0.22 | (0.08, 0.36) | .002 | ** |  | 0.41 | (0.24, 0.58) | <.001 | *** |

*Notes*: * significant at *p* ≤ .05; ** significant at *p* ≤ .01; *** significant at *p* ≤ .001.

Specification (1): pre-intervention period = from January 31, 2010, to the patient-specific day before entering the program; post-intervention period = the time spent in the navigation program.

Specification (2): pre-intervention period = from January 31, 2010, to the patient-specific day before entering the program; post-intervention period = from the patient-specific day of entering the program to January 31, 2014.

Specification (3): pre-intervention period = the time spent in the navigation program shifted two years to the past; post-intervention period = the time spent in the navigation program.

Table C3: Number of emergency room visits

|  | (1) | | | |  | (2) | | | |  | (3) | | | |
| --- | --- | --- | --- | --- | --- | --- | --- | --- | --- | --- | --- | --- | --- | --- |
|  | Est. | 95% CI | *p*-value | |  | Est. | 95% CI | *p*-value | |  | Est. | 95% CI | *p*-value | |
| Intercept | -0.05 | (-0.29, 0.20) | .71 | . |  | -0.05 | (-0.29, 0.20) | .71 | . |  | -0.05 | (-0.29, 0.19) | .70 | . |
| Intervention group | 0.18 | (-0.14, 0.50) | .28 | . |  | 0.18 | (-0.14, 0.50) | .27 | . |  | 0.05 | (-0.30, 0.40) | .79 | . |
| Post period | -0.05 | (-0.27, 0.18) | .69 | . |  | -0.04 | (-0.26, 0.18) | .71 | . |  | -0.04 | (-0.27, 0.18) | .70 | . |
| Intervention group × Post period | 0.24 | (-0.07, 0.54) | .13 | . |  | 0.15 | (-0.15, 0.44) | .33 | . |  | 0.38 | (0.05, 0.71) | .025 | * |

*Notes*: * significant at *p* ≤ .05; ** significant at *p* ≤ .01; *** significant at *p* ≤ .001.

Specification (1): pre-intervention period = from January 31, 2010, to the patient-specific day before entering the program; post-intervention period = the time spent in the navigation program.

Specification (2): pre-intervention period = from January 31, 2010, to the patient-specific day before entering the program; post-intervention period = from the patient-specific day of entering the program to January 31, 2014.

Specification (3): pre-intervention period = the time spent in the navigation program shifted two years to the past; post-intervention period = the time spent in the navigation program.

Table C4: Number of inpatient stays

|  | (1) | | | |  | (2) | | | |  | (3) | | | |
| --- | --- | --- | --- | --- | --- | --- | --- | --- | --- | --- | --- | --- | --- | --- |
|  | Est. | 95% CI | *p*-value | |  | Est. | 95% CI | *p*-value | |  | Est. | 95% CI | *p*-value | |
| Intercept | -0.76 | (-1.00, -0.51) | <.001 | *** |  | -0.76 | (-1.00, -0.51) | <.001 | *** |  | -0.76 | (-1.00, -0.52) | <.001 | *** |
| Intervention group | -0.04 | (-0.37, 0.30) | .82 | . |  | -0.04 | (-0.38, 0.29) | .80 | . |  | -0.16 | (-0.55, 0.23) | .42 | . |
| Post period | -0.07 | (-0.31, 0.17) | .57 | . |  | -0.06 | (-0.30, 0.17) | .60 | . |  | -0.07 | (-0.31, 0.17) | .58 | . |
| Intervention group × Post period | 0.14 | (-0.25, 0.52) | .49 | . |  | 0.05 | (-0.31, 0.42) | .77 | . |  | 0.25 | (-0.20, 0.69) | .28 | . |

*Notes*: * significant at *p* ≤ .05; ** significant at *p* ≤ .01; *** significant at *p* ≤ .001.

Specification (1): pre-intervention period = from January 31, 2010, to the patient-specific day before entering the program; post-intervention period = the time spent in the navigation program.

Specification (2): pre-intervention period = from January 31, 2010, to the patient-specific day before entering the program; post-intervention period = from the patient-specific day of entering the program to January 31, 2014.

Specification (3): pre-intervention period = the time spent in the navigation program shifted two years to the past; post-intervention period = the time spent in the navigation program.

Table D1: Appointment arrivals

|  | (1) | | | |  | (2) | | | |  | (3) | | | |
| --- | --- | --- | --- | --- | --- | --- | --- | --- | --- | --- | --- | --- | --- | --- |
|  | Est. | 95% CI | *p*-value | |  | Est. | 95% CI | *p*-value | |  | Est. | 95% CI | *p*-value | |
| Intercept | 0.22 | (0.07, 0.38) | .006 | ** |  | 0.22 | (0.07, 0.38) | .005 | ** |  | 0.22 | (0.06, 0.38) | .006 | ** |
| Intervention group | -0.10 | (-0.32, 0.12) | .38 | . |  | -0.10 | (-0.32, 0.12) | .38 | . |  | -0.17 | (-0.43, 0.08) | .19 | . |
| Post period | -0.30 | (-0.46, -0.13) | <.001 | *** |  | -0.30 | (-0.46, -0.14) | <.001 | *** |  | -0.29 | (-0.45, -0.13) | <.001 | *** |
| Intervention group × Post period | 0.30 | (0.07, 0.52) | .009 | ** |  | 0.25 | (0.03, 0.46) | .025 | * |  | 0.38 | (0.12, 0.63) | .004 | ** |

*Notes*: * significant at *p* ≤ .05; ** significant at *p* ≤ .01; *** significant at *p* ≤ .001.

Specification (1): pre-intervention period = from January 31, 2010, to the patient-specific day before entering the program; post-intervention period = the time spent in the navigation program.

Specification (2): pre-intervention period = from January 31, 2010, to the patient-specific day before entering the program; post-intervention period = from the patient-specific day of entering the program to January 31, 2014.

Specification (3): pre-intervention period = the time spent in the navigation program shifted two years to the past; post-intervention period = the time spent in the navigation program.

Table D2: Appointment cancellations

|  | (1) | | | |  | (2) | | | |  | (3) | | | |
| --- | --- | --- | --- | --- | --- | --- | --- | --- | --- | --- | --- | --- | --- | --- |
|  | Est. | 95% CI | *p*-value | |  | Est. | 95% CI | *p*-value | |  | Est. | 95% CI | *p*-value | |
| Intercept | -1.30 | (-1.50, -1.09) | <.001 | *** |  | -1.30 | (-1.50, -1.09) | <.001 | *** |  | -1.29 | (-1.5, -1.08) | <.001 | *** |
| Intervention group | 0.04 | (-0.22, 0.30) | .75 | . |  | 0.05 | (-0.21, 0.30) | .73 | . |  | -0.00 | (-0.29, 0.29) | .98 | . |
| Post period | 0.13 | (-0.06, 0.31) | .18 | . |  | 0.13 | (-0.06, 0.32) | .17 | . |  | 0.12 | (-0.07, 0.30) | .21 | . |
| Intervention group × Post period | 0.11 | (-0.14, 0.36) | .39 | . |  | 0.12 | (-0.13, 0.36) | .35 | . |  | 0.15 | (-0.13, 0.43) | .28 | . |

*Notes*: * significant at *p* ≤ .05; ** significant at *p* ≤ .01; *** significant at *p* ≤ .001.

Specification (1): pre-intervention period = from January 31, 2010, to the patient-specific day before entering the program; post-intervention period = the time spent in the navigation program.

Specification (2): pre-intervention period = from January 31, 2010, to the patient-specific day before entering the program; post-intervention period = from the patient-specific day of entering the program to January 31, 2014.

Specification (3): pre-intervention period = the time spent in the navigation program shifted two years to the past; post-intervention period = the time spent in the navigation program.

Table D3: Appointment no-shows

|  | (1) | | | |  | (2) | | | |  | (3) | | | |
| --- | --- | --- | --- | --- | --- | --- | --- | --- | --- | --- | --- | --- | --- | --- |
|  | Est. | 95% CI | *p*-value | |  | Est. | 95% CI | *p*-value | |  | Est. | 95% CI | *p*-value | |
| Intercept | -1.22 | (-1.4, -1.04) | <.001 | *** |  | -1.22 | (-1.40, -1.04) | <.001 | *** |  | -1.22 | (-1.40, -1.05) | <.001 | *** |
| Intervention group | 0.10 | (-0.14, 0.35) | .41 | . |  | 0.10 | (-0.14, 0.35) | .41 | . |  | 0.23 | (-0.06, 0.53) | .12 | . |
| Post period | 0.29 | (0.11, 0.46) | .001 | ** |  | 0.29 | (0.11, 0.46) | .001 | ** |  | 0.29 | (0.12, 0.47) | .001 | ** |
| Intervention group × Post period | -0.54 | (-0.80, -0.29) | <.001 | *** |  | -0.48 | (-0.73, -0.23) | <.001 | *** |  | -0.68 | (-0.97, -0.38) | <.001 | *** |

*Notes*: * significant at *p* ≤ .05; ** significant at *p* ≤ .01; *** significant at *p* ≤ .001.

Specification (1): pre-intervention period = from January 31, 2010, to the patient-specific day before entering the program; post-intervention period = the time spent in the navigation program.

Specification (2): pre-intervention period = from January 31, 2010, to the patient-specific day before entering the program; post-intervention period = from the patient-specific day of entering the program to January 31, 2014.

Specification (3): pre-intervention period = the time spent in the navigation program shifted two years to the past; post-intervention period = the time spent in the navigation program.
